# The Potential Risk Compensation after Receiving HPV Vaccination among Men Who Have Sex with Men in Southwest China: A HAPA-Based Analysis

**DOI:** 10.3390/vaccines11091429

**Published:** 2023-08-28

**Authors:** Zhen Cao, Han Jiang, Wei He, Haiying Pan, Cong Zhang, Xiaoni Zhong

**Affiliations:** School of Public Health, Chongqing Medical University, Chongqing 400016, China; 2021110590@stu.cqmu.edu.cn (Z.C.); 2021110600@stu.cqmu.edu.cn (H.J.); 2020111423@stu.cqmu.edu.cn (W.H.); 2020111428@stu.cqmu.edu.cn (H.P.); 2022120781@stu.cqmu.edu.cn (C.Z.)

**Keywords:** men who have sex with men (MSM), HPV vaccination, risk compensation, health action process approach (HAPA)

## Abstract

*Background*: men who have sex with men (MSM) are a high-risk group for human papillomavirus (HPV) infection, and the HPV vaccine is effective in preventing it. However, according to risk compensation theory, people may increase sexual risk behaviors after receiving HPV vaccination. Based on the Health Action Process Approach (HAPA), this study investigated the influencing factors to predict intention to reduce condom use (risk compensation intention) among MSM after taking HPV vaccination in southwest China. *Methods*: we conducted a cross-sectional study among 948 MSM in southwest China using a non-probability sampling method and an electronic questionnaire including sociodemographic characteristics, sexual risk behavior characteristics, HPV-related prevention behavior, and the HAPA scale. Confirmatory factor analysis was performed using a structural equation model. *Results*: among 948 MSM, the incidence rate of reducing the frequency of condom use was 14.1%. The structural equation model showed that self-efficacy (β = −0.378, *p* = 0.020) and positive outcome expectancy (β = 0.366, *p* < 0.05) had greater effects on behavioral intention, followed by negative outcome expectancy (β = −0.296, *p* < 0.05) and risk perception (β = −0.232, *p* < 0.05). *Conclusions*: risk compensation may not be a major barrier to receiving HPV vaccination among MSM. Nevertheless, the recognition of possible risk compensation is necessary to implement appropriate interventions to reduce the occurrence of risk compensation.

## 1. Introduction

Human papillomavirus (HPV) is one of the most common sexually transmitted diseases (STD) worldwide. At present, more than 200 types of HPV have been identified [1]. Almost all cervical cancers, 90% of anal cancers, and 30% of oropharyngeal cancers worldwide are caused by HPV [2]. HPV infection is common among men who have sex with men (MSM). MSM have significantly higher rates of HPV infection compared to heterosexual men and women [3,4,5]. Meta-analysis showed that the prevalence of HPV infection was very high among MSM internationally (63.9% for HIV-negative MSM and 92.6% for HIV-positive MSM) [6], and the overall HPV prevalence in China was 66.3% [7]. HPV infection can cause genital warts, penile, anal, and oropharyngeal cancers in men [8,9,10]. MSM are at high risk of HPV infection and persistence, carrying multiple HPV types and HPV-related diseases with rapid progression to malignancies [11].

The HPV vaccine is effective in preventing HPV infection and the diseases it causes. The Advisory Committee on Immunization Practices (ACIP) recommends either nine-valent HPV or four-valent HPV vaccines for MSM [12]. Numerous studies have shown that nine-valent HPV or four-valent HPV vaccines are both effective and cost-effective in preventing HPV-related diseases among males [13,14,15]. In 2011, the United States became the first country to incorporate regular HPV vaccination for men, including MSM under the age of 26, into its national vaccination program [16]. By March 2017, 11 countries had provided male vaccination [17]. In China, males are still excluded from the HPV vaccination program [18]. Previous studies have found that MSM had a high willingness for HPV vaccination in China, and more than 80% of MSM were willing to vaccinate; thus, they could be considered for inclusion in the HPV vaccination program [19,20].

However, HPV vaccination has the potential to increase sexual risk behavior while providing benefits to MSM. This phenomenon is called risk compensation. Risk compensation theory refers to the concern that those who undergo a preventive intervention (e.g., HPV vaccination) may engage in behaviors that put them at risk (e.g., reducing condom use) because they feel protected [21]. It has been used to study the sexual behaviors of people at high risk of sexually transmitted infections (STIs) and HIV infection. For example, the introduction of HIV pre-exposure prophylaxis (PrEP), though significantly reducing the risk of HIV transmission, was associated with a decrease in the willingness to use condoms and an increase in the number of sexual partners [22,23]. Therefore, some studies have been concerned with HPV vaccination encouraging sexual risk behaviors, leading to the occurrence of risk compensation [24,25]. At present, studies on risk compensation after taking HPV vaccination mainly focus on women. As an intervention to reduce the risk of STD among MSM, there are few studies on the behavioral intention and influencing factors of risk compensation among MSM after receiving HPV vaccination.

The theoretical framework of this study is the Health Action Process Approach (HAPA) proposed by German psychologist Schwarzer, which is often applied to predict various health behaviors [26,27]. The HAPA is a dual-phase model that distinguishes pre-intentional motivational and post-intentional volitional phases involved in behavior formulation [28]. In the pre-intentional motivational phase, intention is regarded as a primary predictor of behavior. Outcome expectancy (i.e., the belief that the target behavior will lead to effective outcomes for the individual) and self-efficacy (i.e., beliefs in one’s personal capacity to perform the target behavior) are posited as the main factors influencing intention. Risk perception (i.e., the belief in the severity and susceptibility that may result from not performing the target behavior) is posited as a remote antecedent of intention formation. In the volitional phase, there are mainly two parts: (1) action planning and coping plan, and (2) maintenance and recovery self-efficacy. The volitional phase could not be used for this study because MSM do not receive HPV vaccination. It is appropriate to apply the pre-intentional motivational phase of HAPA as the theoretical framework of this study. Some research has also only focused on it [29,30,31]. Previous studies have consistently supported the use of HAPA for vaccination, as all structures of the model predict subsequent vaccination behavior. It has also been applied to explore intentions for influenza vaccination [32], HPV vaccination [24], and COVID-19 vaccination [33].

As more and more countries include males in the HPV vaccination program, it is worth paying attention to whether the psychological cognition and sexual behavior of individuals among MSM will change after receiving HPV vaccination. Therefore, this study investigates the influencing factors to predict risk compensation intention among MSM after taking HPV vaccination in southwest China, and provides evidence for subsequent risk coping strategies of such prevention measures.

## 2. Materials and Methods

### 2.1. Participants and Recruitment

**Figure 1:**
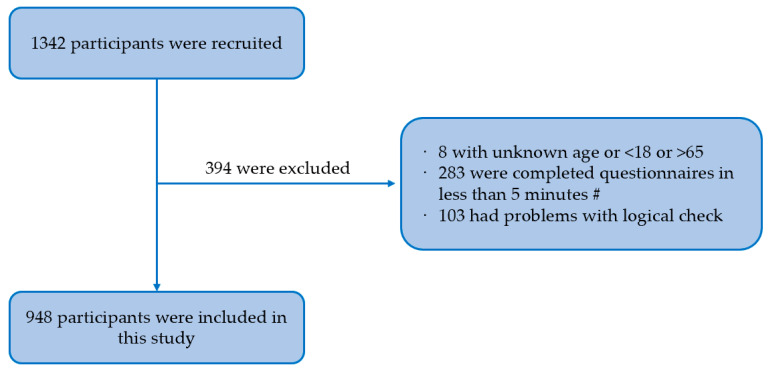
Flow chart of participants selection. Note: #—after testing, we assessed that participants should have completed the questionnaire in at least 5 min.

From October to November 2022, a non-probability sampling method (including collaboration with non-governmental organizations (NGOs), peer recommendations, and core members (“snowball”)) was used to recruit participants in two cities in Chongqing and Sichuan, China. The inclusion criteria for MSM were as follows: (1) between the ages of 18 and 65 years; (2) self-reported anal sex with one or more male partners in the last year; (3) no serious mental illness or intellectual defect. This study was conducted in an anonymous cross-sectional online survey. Some participants were recruited from the MSM cohort established in the previous study (a cohort study of the National Science and Technology Major Project, registration number: 2018ZX10721102-005), whereas others were recruited from other NGOs. The investigators distributed electronic questionnaires to participants after acquiring their informed consent. If participants did not agree to continue, they had the right to terminate the survey at any time. After completing the questionnaire and passing the review, each participant received a reward of 5 RMB (approximately $0.73). This study was approved by the Ethics Committee of Chongqing Medical University (2019001, 28 May 2019). A total of 1342 participants were recruited for the study. A total of 394 MSM (29.4%) were excluded for the reasons displayed in Figure 1, and the remaining 948 participants met the study criteria.

### 2.2. Measurements

#### 2.2.1. Background Characteristics

The information collected included the following four parts: (1) sociodemographic characteristics (i.e., age, household registration, ethnicity, educational level, employment status); (2) characteristics of sexual risk behaviors in the past six months (i.e., number of male partners, condom use during anal sex with male partners); (3) HPV-related preventive behavior (HPV counseling); (4) HPV vaccine-related intentions.

#### 2.2.2. HAPA Scale

Based on the HAPA model, MSM were asked about their views on condom use in a hypothetical situation of having received HPV vaccination. On the basis of the previous literature, this scale consisted of 14 items, including four constructs (risk perception, positive outcome expectancy, negative outcome expectancy, and self-efficacy). The scale was scored according to the Likert five-point scoring method (Table 1).

**Figure 2:**
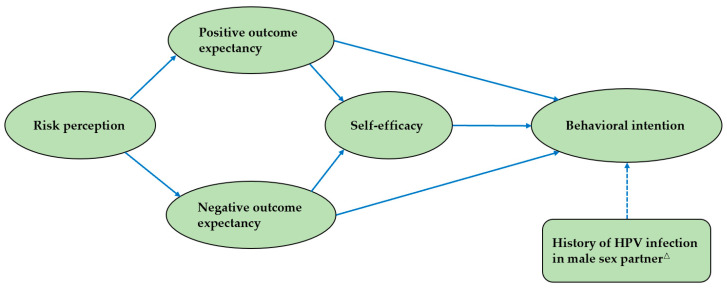
The initial structural equation model of HAPA. Note: the solid line indicates the theoretical model. The dotted line indicates the added path for this study. △ indicates whether a male partner has been infected with HPV in the last six months.

The risk perception consisted of four items, with a higher score indicating a perceived greater increase in the risk of HPV infection. There were two items that made up the positive outcome expectancy, wherein a higher score meant that the perception of reducing condom use after HPV vaccination would result in a more positive outcome. The negative outcome expectancy consisted of three items, with a higher score suggesting that the perceived outcome of reducing condom use after getting HPV vaccine would lead to a more negative impact. The self-efficacy part comprised four items, with a higher score indicating greater confidence in consistently using condoms after receiving the HPV vaccine. Behavioral intention was measured by asking “How condom use may change when you have anal sex with your male sex partner after receiving HPV vaccination”. (1 = very likely to increase, 2 = likely to increase, 3 = remain the same, 4 = likely to reduce, 5 = very likely to reduce). The answers “likely to reduce” and “very likely to reduce” were regarded as the behavioral intention to generate risk compensation. The initial structural equation model of HAPA is displayed in Figure 2.

### 2.3. Statistical Analysis

SAS version 9.4 was used for data collation and descriptive analysis. Categorical data were described in frequencies and percentages. Structural equation modeling was performed to construct HAPA models using a weighted least squares mean and variance adjusted estimator. Mplus version 8.3 (Los Angeles, LA, USA: Asparouhov & Muthén, 2019) was used to evaluate the factor structure and the relationship of relative variables. Cronbach’s alpha and composite reliability (CR) were used to measure reliability, both of which needed to be greater than 0.70, indicating good internal consistency. The average variance extracted (AVE) was used to measure the convergent validity, and a value greater than 0.50 indicated good convergence. The discriminant validity was considered acceptable if the correlation coefficient between structures was lower than the square root of the corresponding structure. Model fit was assessed using the chi-squared and degree of freedom ratio (χ^2^/df), comparative fit index (CFI), Tucker Lewis index (TLI), standardized root mean square residual (SRMR), and root mean square error of approximation (RMSEA). The model fitting was acceptable when χ^2^/df < 5, CFI > 0.90, TLI > 0.90, SRMR < 0.08, and RMSEA < 0.08. Statistical significance was considered at *p* < 0.05.

## 3. Results

### 3.1. Background Characteristics

A total of 948 MSM were included in this study, of which 59.8% were aged 18–27, 59.7% were urban, 95.7% were Han, 54.6% had a bachelor’s degree or above, 55.1% were employed, 83.5% were married, and 72.7% had a monthly salary of less than 5000 RMB. In terms of the characteristics of sexual risk behaviors in the past six months, 33.3% of MSM had two or more male sex partners, whereas only 48.5% had used condoms every time when having anal sex with their male partners. A total of 2.1% had a male partner who had been infected with HPV, and 3.4% had a history of STD. In terms of HPV-related preventive behavior, 41.5% had HPV counseling. In addition, 87.6% indicated that they were very willing or willing to receive HPV vaccination. If receiving HPV vaccination, 14.1% of MSM said they were very likely or likely to reduce condom use (risk compensation intention) (Table 2).

### 3.2. Measurement Model

The initial measurement model was tested. Table 3 describes the mean, standard deviation, factor loading, CR, AVE, and Cronbach’s alpha of each construct. The factor loading of each item was greater than 0.5, which was at an acceptable level and statistically significant (*p* < 0.001). CR and Cronbach’s alpha were greater than 0.7, indicating good internal consistency of the data. AVE was greater than 0.5, indicating good convergence validity. At the same time, the square root of AVE was greater than other correlation coefficients, indicating that the discriminant validity was acceptable (Table 4). Confirmatory factor analysis was performed using a structural equation model. The model fit indices were as follows: χ^2^/df = 4.686, CFI = 0.990, TLI = 0.988, SRMR = 0.042, and RMSEA = 0.062. These fit indices met the recommended values and indicated that the model was acceptable. Therefore, the model established could be considered acceptable.

### 3.3. Structural Model

**Figure 3:**
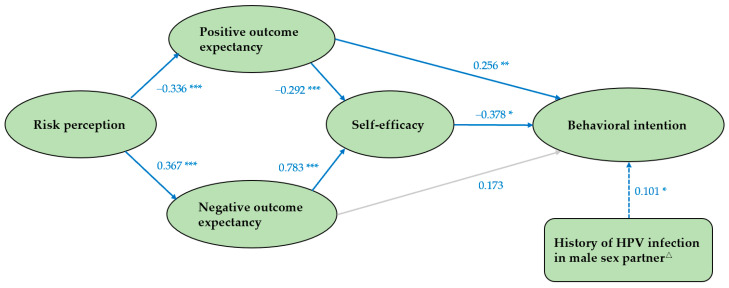
The path diagram of structural model. Note: the solid line indicates the theoretical model. The dotted line indicates the added path for this study. The gray line indicates that the coefficient is not statistically significant and the pathway is not valid. △ indicates whether a male partner has been infected with HPV in the last six months. * *p* < 0.05, ** *p* < 0.01, *** *p* < 0.001.

Structural model analysis showed (Figure 3) that self-efficacy and positive outcome expectancy had a greater effect on risk compensation intention, followed by negative outcome expectancy and risk perception. Positive outcome expectancy (β = 0.256, *p* < 0.001), self-efficacy (β = −0.378, *p* = 0.020), and history of HPV infection in male sex partner (β = 0.101, *p* = 0.038) had direct effects on behavioral intention, whereas negative outcome expectancy (β = −0.296, *p* < 0.05) and risk perception (β = −0.232, *p* < 0.05) had indirect effects on it. What is inconsistent with the hypothesized model is that negative outcome expectancy had no direct effect on behavioral intention (*p* = 0.242). Positive outcome expectancy was positively correlated with behavioral intention, whereas self-efficacy, risk perception, and negative outcome expectancy were negatively correlated with it.

Our study found that self-efficacy played an indirect role in the relationship between positive outcome expectancy and behavioral intention. The direct effect of positive outcome expectancy on behavioral intention was 0.256 (*p* = 0.001), and the indirect effect was 0.110 (*p* < 0.05) and the total effect was 0.366 (*p* < 0.05), in which 30.1% of the effect of positive outcome expectancy on behavioral intention was caused by self-efficacy. The significant standardized path coefficients of the structural equation model are depicted in Table 5.

## 4. Discussion

HPV is one of the most common sexually transmitted diseases worldwide. It is strongly associated with a variety of human cancers, including cervical, penile, anal, and oral cancers. Co-factors (e.g., environmental carcinogens, cigarette smoking, immunosuppression) are deemed to have an effect on contributing to HPV-related carcinogenesis recognized by the International Agency for Research on Cancer (IARC) [35,36,37]. In males, the development of precancerous lesions is a growing problem, especially among MSM. HPV vaccination is an important measure to reduce the precancerous and cancerous lesions associated with HPV infection [38]. It has a good immune response and can play a direct protective role in men. Current vaccines are able to cover most viral subtypes of HPV-related diseases.

However, risk compensation theory predicts that the emergence of biomedical prevention strategies may be accompanied by an increase in sexual risk behaviors. There were some direct links between risk compensation and vaccines [39,40,41]. The potential risk compensation after receiving HPV vaccination, as an intervention to reduce the risk of STD among MSM, is a topic of concern. Based on the HAPA, this study investigated the influencing factors to predict risk compensation intention among MSM after taking HPV vaccination in southwest China. The structural equation model was used for confirmatory factor analysis. Overall, the model fits well.

This study found that 87.6% of MSM were willing to receive HPV vaccination, which is similar to the results of previous studies in China (86.2% and 82.8%) [19,20]. It can be seen that MSM had a high willingness to receive HPV vaccination. Risk compensation intention was investigated among all participants in a hypothetical situation of having received HPV vaccination. We only studied one form of risk compensation (changes in condom use intention). Theoretically, MSM might increase the number of sex partners while reducing the frequency of condom use. Such cases may just be rare occurrences [42]. The results showed that 14.1% of MSM would reduce their intention to use condoms after taking HPV vaccination. The incidence rate of risk compensation intention investigated in our study was higher than the previous study among MSM in Hong Kong (8.0%) [24], but lower than those related to some other biomedical prevention measures among men who have sex with men, such as voluntary medical male circumcision (15.9%) [43] and HIV vaccines (34.6%) [44]. This study suggests that risk compensation may not be a major barrier to receiving HPV vaccination among MSM. Nevertheless, the recognition of possible risk compensation is necessary to implement appropriate interventions for reducing the occurrence of risk compensation after HPV vaccination.

Structural equation model analysis showed that self-efficacy and positive outcome expectancy had a greater effect on behavioral intention, followed by negative outcome expectancy and risk perception. Risk perception was negatively correlated with risk compensation intention, that is, the reduced risk perception of HPV in MSM may lead to an increased willingness to risk compensation after taking HPV vaccination, which is similar to the results of previous studies on risk compensation behavior [42,45]. Our finding on the relationship between risk perception and risk compensation intention also confirmed the risk compensation theory. After taking HPV vaccination, MSM’s perceived risk may decrease, thereby increasing sexual risk behavior and generating risk compensation. Therefore, when promoting HPV vaccination, we should take care to remind MSM that the protective effect of the HPV vaccine is limited and it should not be used as a substitute for condom use, but rather as a complementary strategy to prevent STDs.

Positive outcome expectancy was one of the main influencing factors of risk compensation intention and was positively correlated with it. After receiving HPV vaccination, MSM believed that condomless anal intercourse would increase intimacy and trust between them and their male sex partners. Such a positive outcome expectancy was related to a higher intention to conduct risk compensation behavior. Several studies have also paid attention to the effect of intimacy and trust on sexual risk behavior among MSM. Condomless anal intercourse was seen by MSM as a bond to build a trusting relationship, and more than half of the participants cited it as a reason for not using condoms [46]. A quantitative study on MSM found that there was a relationship between relationship length, trust, and condom use. If the relationship lasted longer, the increase of trust would lead to a decrease in condom use [47]. Negative outcome expectancy had a negative effect on behavioral intention, and increasing it might reduce risk compensation. Based on this, future interventions should emphasize negative outcomes (e.g., condomless anal intercourse can give you HPV/other STDs.) and should improve the negative outcome expectancy of MSM to prevent the occurrence of risk compensation after HPV vaccination in this population.

Self-efficacy had a direct negative effect on risk compensation intention, and increasing self-efficacy may reduce risk compensation. Self-efficacy of condom use refers to how confident people are in their ability to use condoms. Previous studies have shown that lower self-efficacy of condom use was strongly associated with higher sexual risk behaviors [48,49,50], and our study confirmed this relationship. This finding highlights the importance of finding creative, effective ways to improve self-efficacy of condom use among MSM and suggests that achieving this goal may help reduce the likelihood of risk compensation after receiving HPV vaccination among MSM. Combined with the above negative correlation results, it is suggested that when promoting HPV vaccination to MSM, we should enhance the risk awareness of MSM, emphasize the negative outcome expectancy of condom use reduction, improve the self-efficacy of condom use, and decrease the positive outcome expectancy of condom use reduction to reduce the occurrence of risk compensation.

There are some limitations to the current study. First, our study is a cross-sectional survey, which limited causal inference. Cohort studies are needed to confirm these findings in the future. Second, MSM do not receive HPV vaccination. We investigated the risk compensation intention in a hypothetical situation of having received HPV vaccination. Although behavioral intention is deemed to be a major factor affecting actual behavior, some differences occur between actual behavior and intention. Third, due to the obscuring nature of the MSM, a non-probabilistic sampling method was used in this study. The results may be biased, which may limit the generalization of our findings to other MSM populations.

## 5. Conclusions

The HAPA model could be used to predict the risk compensation intention after taking HPV vaccination among MSM in southwest China, in which self-efficacy and positive outcome expectancy are the important predictor factors. Risk compensation may not be a major barrier to receiving HPV vaccination among MSM. Nevertheless, it is necessary to acknowledge possible risk compensation. Future research should focus on improving MSM’s risk perception of HPV, emphasize the negative outcome expectancy of condom use reduction, decrease the positive outcome expectancy of condom use reduction, and find effective ways to improve the self-efficacy of condom use among MSM, aiming to reduce the occurrence of risk compensation after HPV vaccination and providing interventions for subsequent risk coping strategies of such prevention measures.

## Figures and Tables

**Table 1:** The HAPA scale and value assignment.

Constructs	Value Assignment
Risk perception RP1 How likely you think HPV infection is to cause anal, penile, or oral cancer. RP2 HPV infection increases the risk of HIV infection. RP3 Genital warts can be a serious hazard to your health. RP4 Cancer of the anus, penis, or mouth can cause serious harm to your health.	1 = very small, 2 = small, 3 = average, 4 = large, 5 = very large 1 = strongly disagree, 2 = disagree, 3 = neutral, 4 = agree, 5 = strongly agree 1 = strongly disagree, 2 = disagree, 3 = neutral, 4 = agree, 5 = strongly agree
Positive outcome expectancy POE1 Condomless anal intercourse will increase intimacy between you and your male sex partner. POE2 Condomless anal intercourse will increase trust between you and your male sex partner.	1 = strongly disagree, 2 = disagree, 3 = neutral, 4 = agree, 5 = strongly agree
Negative outcome expectancy NOE1 Condomless anal intercourse can give you HPV. NOE2 Condomless anal intercourse can give you other STDs. NOE3 Condomless anal intercourse will make you worried.	1 = strongly disagree, 2 = disagree, 3 = neutral, 4 = agree, 5 = strongly agree
Self-efficacy SE1 You are confident to use condoms consistently no matter what your condition is. SE2 You are confident to use condom consistently even when your sex partner is not willing to use it. SE3 Even if you want to be intimate with your sex partner, you have the confidence to use condom consistently. SE4 You are confident that you can suggest condom use without your sex partner thinking that you are worried about him getting an STD.	1 = strongly disagree, 2 = disagree, 3 = neutral, 4 = agree, 5 = strongly agree
Behavioral intention BI How condom use may change when you have anal sex with your male sex partner after receiving HPV vaccination.	1 = very likely to increase, 2 = likely to increase, 3 = remain the same, 4 = likely to reduce, 5 = very likely to reduce

Note: RP, risk perception; POE, positive outcome expectancy; NOE, negative outcome expectancy; SE, self-efficacy; BI, behavioral intention; STDs, sexually transmitted diseases.

**Table 2:** Background characteristics of MSM in southwest China (*N* = 948).

Variable	*N*	%	Variable	*N*	%
**Sociodemographic characteristics**			**Sexual behavior characteristics**		
**Age**			**(in the past six months)**		
18–27	567	59.8	**Number of male partners**		
28–45	310	32.7	0	217	22.9
>45	71	7.5	1	415	43.8
**Household registration**			≥2	316	33.3
Urban areas	566	59.7	**Condom use when having anal sex with a male partner**		
Rural areas	382	40.3	Every time	460	48.5
**Ethnicity**			Sometimes	140	14.8
Han nationality	907	95.7	Rarely	62	6.5
Other ethnic minorities	41	4.3	No anal sex	286	30.2
**Educational level**			**Whether had male sex partners who had been infected with HPV**		
Junior high and below	59	6.2	No	720	76
High school	158	16.7	Unclear	208	21.9
Junior college	213	22.5	Yes	20	2.1
College and above	518	54.6	**Whether had been diagnosed with an STD by a doctor**		
**Employment status**			No	916	96.6
Employed	522	55.1	Yes	32	3.4
Retired or unemployed	96	10.1	**HPV-related preventive behavior ^#^**		
Students	330	34.8	**HPV counseling**		
**Marital status**			Done	393	41.5
Unmarried	792	83.5	Never	555	58.5
Married	80	8.4	**HPV vaccine-related intentions**		
Divorced/widowed	76	8.0	**Whether were willing to receive HPV vaccine**		
**Personal monthly income**			very unwilling or unwilling	17	1.8
1000 RMB or less	139	14.7	uncertain	102	10.8
1001~3000 RMB	317	33.4	very willing or willing	829	87.5
3001~5000 RMB	233	24.6	**How condom use may change when having anal sex with male sex partner after receiving HPV vaccination**		
5001~10,000 RMB	195	20.6	very likely or likely to increase	251	26.5
10,000 RMB or more	64	6.8	remain the same	563	59.4
			very likely or likely to reduce	134	14.1

Note: ^#^ in China, due to the lack of HPV screening guidelines for MSM and other restrictions that prevent MSM from going to hospitals for HPV screening, few MSM have been tested [34]; thus, HPV testing was not included in the questionnaire in this study.

**Table 3:** Descriptive statistics, factor loading, convergent validity, and reliability analysis.

Construct	Mean	SD	Factor Loading	CR (>0.7)	AVE (>0.5)	Cronbach’s Alpha
RP	3.637	0.784	0.578–0.863	0.836	0.567	0.766
POE	2.613	1.154	0.929–0.931	0.928	0.865	0.899
NOE	3.817	0.846	0.733–0.909	0.889	0.729	0.830
SE	3.922	0.865	0.863–0.931	0.949	0.823	0.930
BI	2.759	0.999	-	-	-	-

Notes: RP, risk perception; POE, positive outcome expectancy; NOE, negative outcome expectancy; SE, self-efficacy; BI, behavioral intention. SD, standard deviation; CR, composite reliability; AVE, average variance extracted.

**Table 4:** Discriminant validity.

	RP	POE	NOE	SE	BI
RP	**0.753**				
POE	−0.336 ***	**0.930**			
NOE	0.367 ***	−0.123 ***	**0.854**		
SE	0.386 ***	−0.389 ***	0.819 ***	**0.907**	
BI	−0.168 ***	0.382 ***	−0.168 **	−0.336 ***	—

Note: the bold fonts in the leading diagonals are the square root of AVEs. Off-diagonal elements are correlations among constructs. RP, risk perception; POE, positive outcome expectancy; NOE, negative outcome expectancy; SE, self-efficacy; BI, behavioral intention. ** *p* < 0.01; *** *p* < 0.001.

**Table 5:** Standardized direct, indirect, and total effects of the final structural equation model.

Path	Standardized Direct Effect	Standardized Indirect Effect	Standardized Total Effect
Risk perception → Behavioral intention	-	−0.232 *	−0.232 *
Positive outcome expectancy → Behavioral intention	0.256 *	0.110 *	0.366 *
Negative outcome expectancy → Behavioral intention	-	−0.296 *	−0.296 *
Self-efficacy → Behavioral intention	−0.378 *	-	−0.378 *
History of HPV infection in male sex partner → Behavioral intention	0.101 *	-	0.101 *

Note: * *p* < 0.05.

## Data Availability

The data involved in the current study are available from the corresponding author on reasonable request.
